# Ecotoxicological evaluation of surface waters in Northern Namibia

**DOI:** 10.1007/s10661-024-12613-2

**Published:** 2024-04-17

**Authors:** L Faulstich, S Wollenweber, Ch Reinhardt-Imjela, R Arendt, A Schulte, H Hollert, S Schiwy

**Affiliations:** 1 Freie Universität Berlin, Berlin, Germany; 2 Goethe-Universität Frankfurt, Frankfurt, Germany

**Keywords:** Effect-based bioassays, Acute toxicity, Mutagenicity, Surface waters, Iishana system, Namibia

## Abstract

The increasing pressure on freshwater systems due to intensive anthropogenic use is a big challenge in central-northern Namibia and its catchment areas, the Kunene and the Kavango Rivers, and the Cuvelai-Etosha Basin, that provide water for more than 1 million people. So far, there is no comprehensive knowledge about the ecological status and only few knowledge about the water quality. Therefore, it is crucial to learn about the state of the ecosystem and the ecological effects of pollutants to ensure the safe use of these resources. The surface waters of the three systems were sampled, and three bioassays were applied on three trophic levels: algae, daphnia, and zebrafish embryos. Additionally, in vitro assays were performed to analyze mutagenicity (Ames fluctuation), dioxin-like potential (micro-EROD), and estrogenicity (YES) by mechanism-specific effects. The results show that acute toxicity to fish embryos and daphnia has mainly been detected at all sites in the three catchment areas. The systems differ significantly from each other, with the sites in the Iishana system showing the highest acute toxicity. At the cellular level, only weak effects were identified, although these were stronger in the Iishana system than in the two perennial systems. Algae growth was not inhibited, and no cytotoxic effects could be detected in any of the samples. Mutagenic effects and an estrogenic potential were detected at three sites in the Iishana system. These findings are critical in water resource management as the effects can adversely impact the health of aquatic ecosystems and the organisms within them.

**Supplementary information:**

The online version contains supplementary material available at 10.1007/s10661-024-12613-2.

## Introduction

Freshwater ecosystems provide important ecosystem services and are biodiversity hot spots (Balian et al., 2008; Stendera et al., 2012). Anthropogenic stressors, such as nutrient enrichments, the discharge of toxic metals (Leal et al., 2016), or organic substances (Christensen et al., 2022), put increasingly high pressure on ecosystems (Jansen et al., 2011). Due to the pollution of surface waters, rivers, and lakes are threatened to lose their biodiversity and are in danger of no longer being able to provide their ecosystem services (Malaj et al., 2014; Vörösmarty et al., 2010). In addition to chemical analysis of water, bioassays are an essential component of water assessment (Di Paolo et al., 2016; Keddy et al., 1995; Methneni et al., 2021) as effect-based bioassays map the effects of the complex sample regardless of the concentration. The harmful concentrations of substances in different organisms (bacteria, green algae, small crustaceans, and fish) are studied to determine aquatic toxicity (Chapman, 2002; Neale et al., 2017). Several studies investigate aquatic systems for ecotoxicological effects (Aragaw & Mekonnen, 2021; Ford et al., 2021; Grund et al., 2011; Hollert et al., 2005; Krein et al., 2012). Most of the studies analyzed aquatic systems of rivers, where active point sources can be identified (Keiter et al., 2006; Pawlowski et al., 2004; Wolf et al., 2022). In addition, it is known that pollutants can induce cytotoxic, endocrine, and mutagenic effects in aquatic systems (Shuliakevich et al., 2022a; Wolf et al., 2022).

In southern Africa, the seasonal precipitation and high evaporation cause a challenging water supply. However, there is still a huge research gap on the state of freshwater ecosystems, although they are ecologically important and diverse (Schoenfuss et al., 2022). The region in southern Angola and central-northern Namibia has an increased incident human water security threat (Vörösmarty et al., 2010). In central-northern Namibia, the landscape is characterized by the Cuvelai-Etosha Basin (CEB), and the Kunene River and Kavango River basins. These three basins are very important for the local water supply but have not been well investigated concerning the potential ecotoxicity of freshwater systems, there are hardly any data on environmental pollution. It is necessary to analyze the current state of the freshwater systems to take appropriate protection or renaturation measures. The intensively used surface waters of these three basins are exposed to many influencing factors, such as water withdrawal for use as potable water, irrigation water, or drinking water for livestock, input of agricultural runoff, and surface runoff from farmland.

In southern Africa, (eco)toxicological investigations have only been used since the 1990s (Wepener & Chapman, 2012). Potential toxic effects on selected organisms were studied for drinking water (Grabow et al., 1991) or to investigate the consequences of mining activities (Connell et al., 1991). In Namibia, endocrine disruption compounds (EDCs) were investigated in water resources close to densely populated areas (Faul et al., 2013, 2014). However, to our current knowledge no studies on ecotoxicological investigations at the Kavango and the Kunene Rivers were conducted. The Kunene River is the subject of several studies concerning river ecology, water governance, and water management (Hipondoka et al., 2018; Meissner & Jacobs, 2016; Schwieger, 2019; Schwieger, 2020). The Kavango River is investigated for discharge, precipitation variability, hydrobiology, and ecology (Bauer-Gottwein et al., 2015; Benitez et al., 2022; Cronberg et al., 1995; Gaughan & Waylen, 2012; Jury, 2009; Kgathi et al., 2006). Gwenzi and Chaukura (2018) described a lack of ecotoxicological studies in African aquatic systems and the resulting dangers of organic contaminants (OCs), estrogenicity, and acute toxicity. In particular, in Sub-Saharan Africa, except South Africa, investigating OCs in potential drinking water is an important task that has not yet been solved satisfactorily. In general, ecotoxicological studies in ephemeral systems in arid and semi-arid regions are rare (Lahr, 1997). In previous studies, the water quality and microplastic pollution of the Iishana were investigated (Faulstich et al., 2022 & 2023). The Iishana have high turbidity values, up to 1615.4 NTU, and elevated salt concentrations, Na up to 429.0 mg/l and Cl up to 678.0 mg/l. Aluminum and iron were found in elevated concentrations (up to 19.3 mg/l Al and 3.9 mg/l Fe^2+^), particularly in suspended solids and sediments (Faulstich et al., 2023). With this composition, the water is not suitable as potable water and must first be treated. With appropriate treatment, the water quality for humans can be achieved, but no sustainable improvement in the condition of the ecosystem is obtained. Parts of the local population are not able to consume treated water for financial reasons. They are, therefore, dependent on the water of the Iishana. Thus, the ecosystem of the Iishana must be considered to determine how its condition can be improved. Poor water quality due to pollutants can directly or indirectly have harmful effects on these organisms and ultimately affect the entire aquatic ecosystem. Ecotoxicological testing methods can be used to identify pollutants and investigate the extent to which they can be taken up and accumulated by organisms. In order to ensure the safe use of water bodies in the long term and to take appropriate treatment measures, their ecological condition must be examined.

This is the first study that investigates the Iishana ecosystem using chemical parameters and ecotoxicological test methods. The water bodies of the Iishana represent an important water resource in the region and, therefore, need to be studied intensively. Thus, this study first gives insight into the hazard potential of the surface waters of the Iishana, Kunene, and Kavango Rivers on organisms and the environment. The main question is if there are any acute toxic or mechanism-specific potential (cytotoxicity, endocrine activity, mutagenicity or dioxin-like potential) in the surface waters of the three basins. After a single application on organisms, the acute toxicity with a toxic effect was measured on three trophic levels: algae, daphnia, and fish embryos. These assays were applied to gain knowledge of the ecotoxicological potential of the surface waters in northern Namibia. Without this data, a final assessment of the waters is not possible. The data obtained in this study can help to identify sources of pollution and take appropriate measures to protect the three investigated ecosystems.

## Methods

### Study area

The Iishana system in central-northern Namibia is a complex hydrological system of drainless depressions and small channels (Iishana, singular Oshana). The Iishana have different sizes, with a depth of 1–7 m (Mendelsohn et al., 2013). When there is enough rainfall, they connect to form a network of narrow drainage ways (Arendt et al., 2021; Faulstich et al., 2022). The Iishana system is part of the Cuvelai-Etosha Basin (CEB) that is neighbored by the Kunene basin in the west and the Kavango basin in the east (Mendelsohn et al., 2013). While ephemeral rivers dominate the endorheic CEB, the Kunene and the Kavango basins are dominated by their namesake perennial rivers that drainage into the Atlantic (Kunene River) and the Okavango Delta (Kavango River) (Meissner & Jacobs, 2016; Steudel et al., 2013). The Kunene River has its source in southwestern Angola, flows to the south, and forms the border between Angola and Namibia. With a discharge of 15 km^3^/year and a difference in altitude of about 1700 m, it is suitable for hydroelectricity (Meissner & Jacobs, 2016). The Kavango River originates in the Angolan highlands, flows southwards, and terminates in the Okavango Delta (McCarthy & Ellery, 1998). In Namibia, the Cubango and Cuito rivers join, build a wetland strip, and form the Okavango River, which is also called the Kavango River. At the border between Angola and Namibia, the discharge is about 10 km^3^/year (Steudel et al., 2013).

Due to the intensive dry (April to September) and rainy seasons (October to March), the water supply is challenging. In the last decade, rainfall was below average in the rainy seasons, resulting in the Iishana not carrying enough water to serve as a water source throughout the dry period (NOAA, 2021). Almost half of the Namibian population lives in the Namibian part of the CEB. Water resources are the freely accessible Iishana and the Calueque-Oshakati canal as part of the public water supply that transports water from Calueque (Angola) to Oshakati (Namibia) (Shuuya & Hoko, 2014).

### Sampling and extraction

**Fig. 1:**
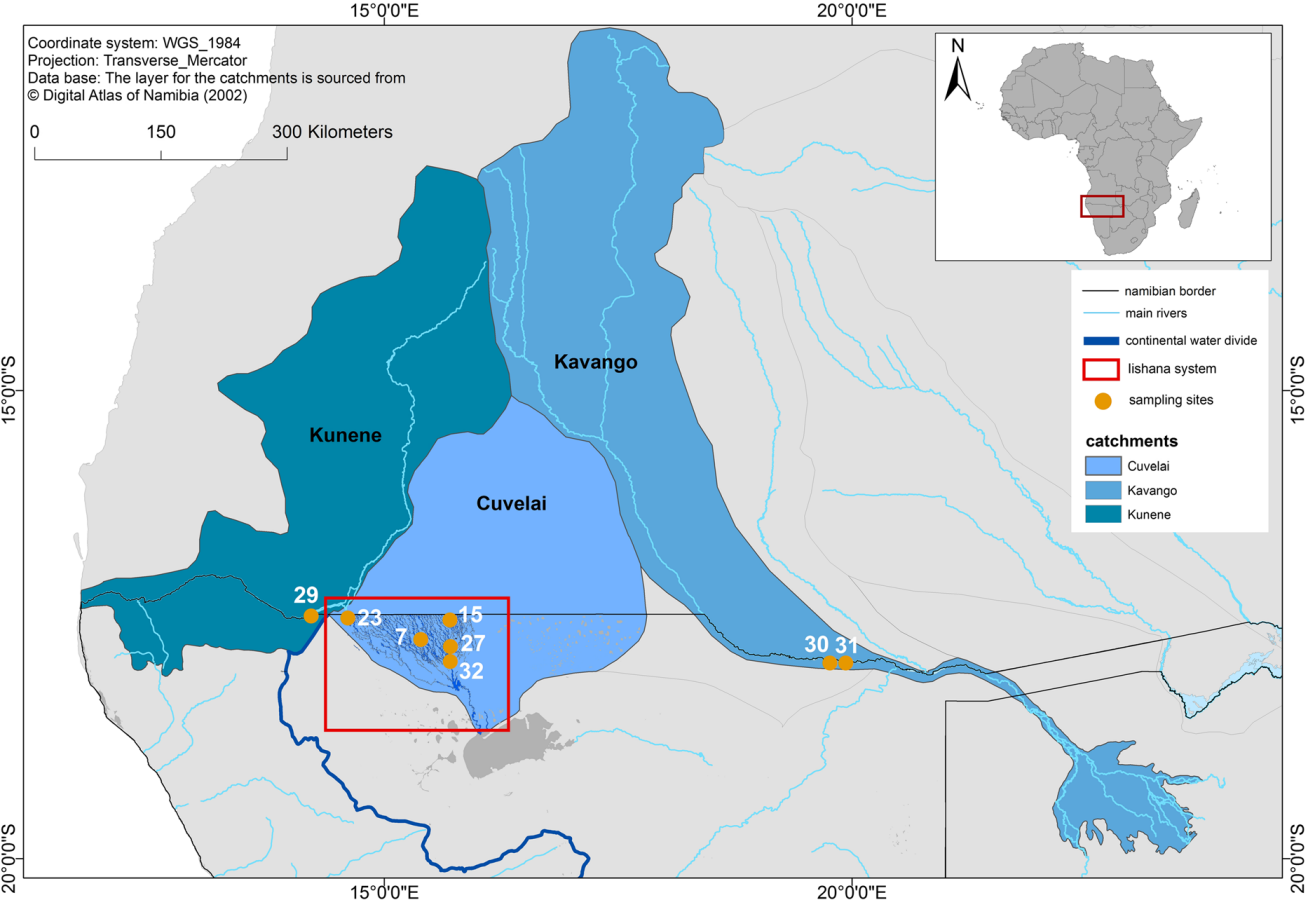
Catchment areas of the three systems with sampling sites and numbers

The Iishana, the Kunene, and the Kavango River water samples were taken in 2019 (Fig. 1 shows the catchment areas, sampling locations, and sample numbers; Supplementary Data 1 includes detailed information of the sampling sites). At every site, several aluminum bottles (4 × 60 ml, 8 × 120 ml, and 1 × 600 ml; Bürkle, Germany) were three times pre-rinsed with Acetone (Acetone, HPLC Grade; CarlRoth, Germany) and filled with water samples. Samples were consistently refrigerated at 4 °C until analysis.

For sample preparation, the individual water samples (1.8 l each) were filtrated with suction through a 0.2-µm fiberglass filter (MN GF-2; Macherey–Nagel, Germany). The samples were concentrated by solid phase extraction (SPE) to extract the per- and poly-fluoroalkyl substances. At first, the columns (CHROMABOND HLB; 60 µm, 15 ml/500 mg; pore diameter 63 Å, particle size 53 µm; Macherey–Nagel, Germany) were conditioned with dichloromethane (Dichloromethane, ROTISOLV HPLC Grade; CarlRoth, Germany), methanol (Methanol, MeOH, HPLC Grade; CarlRoth, Germany), and ultrapure water. Then, the SPE columns were loaded with the samples under a vacuum (− 0.3 – − 0.4 bar). The columns were eluted with 6 ml MeOH and 6 ml DCM. As a solvent keeper, 50 µl dimethylsulfoxide (Dimethylsulfoxide, DMSO, 99.8%; CarlRoth, Germany) was added. The samples were rotated to a minimum (40°C, slowly decreasing pressure) and transferred to amber glass vials. The vials were evaporated under nitrogen to exclude the remaining MeOH.

The resulting extract of 1 ml is 2000-fold concentrated. The water extracts included a realistic environmental matrix of the water body and were frozen for further bioassay testing. A process control (ProCo) of ultrapure water was treated and tested like a sample to control the purity of the extraction.

### Bioassays

In Table 1, the key data of the applied bioassays with the information on endpoints, model organisms, exposure time and vessels, medium quantities, and followed guidelines are shown.

**Table 1:** Key data of the bioassays performed

Bioassay	Method title	Endpoint	Model organism	Exposure duration [h]	Exposure vessels	Medium per vessel [ml]	Guideline
FET test	Fish embryo acute toxicity test	Fish embryo lethality and occurrence of morphological sublethal endpoints	*Danio rerio*	120	24-well plates*	2	ISO 15088:2008 (DIN EN ISO, 2008) OECD Test No. 236 (OECD, 2013a, 2013b)
Daphnia test	Daphnia acute immobilization test	Immobilization of daphnids	*Daphnia magna*	48	glass tubes	10	OECD Test No. 202 (OECD, 2004)
Algae test	Algal growth inhibition test	Growth inhibition	*Raphidocelis subspicata (formerly: Pseudo-kirchneriella subcapitata)*	72	96-well plates*	2	OECD Test No. 201 (OECD, 2011)
Ames assay	Ames fluctuation assay	Induction of reverse mutations	*Salmonella strains* TA100 *and* TA98	48	24-/384-well plates*	0.5 (+ 2.5)/0.05	ISO 11350:2012–05 (DIN EN ISO, 2012), Reifferscheid et al., 2011
Micro-EROD assay	Micro-EROD bioassay	Cytochrome activities	*Rat hepatoma cell line H4IIE*	72	96-well plates*	0.1	Schiwy et al., 2015a
YES assay	Yeast estrogen screening assay	Estrogen receptor binding activity	*Recombinant* *yeast cells*	18–72	96-well plates*	0.2	ISO 19040–1: 2018 (DIN EN ISO, 2018), Routledge & Sumpter, 1996

^*^Starlab International GmbH, Germany

### Fish embryo toxicity test

The zebrafish used were originally from the strain of the Westaquarium (Bad Lauterberg, Germany) and were bred in the Department of Evolutionary Ecology and Environmental Toxicology, Goethe University Frankfurt am Main (Germany). The fish embryo acute toxicity test was applied as a limit test with fertilized eggs of the zebrafish (*Danio rerio)*, based on the DIN guideline and the OECD guideline 236 (DIN EN ISO, 2008; OECD, 2013a; Shuliakevich et al., 2022b). As described in Johann et al. (2021), the experiments were terminated shortly before 120-h post-fertilization (hpf). According to the Directive 2010/63/EU (European Union, 2010), testing zebrafish embryos and larvae before 120 hpf does not require animal ethics test approval. After testing, all embryos were euthanized with a benzocaine-ethanol solution (40 g/ml in ethanol). All samples were tested in the highest concentration (Ref 2) of the water sample extracts of the Iishana and the perennial rivers. For each sample, ten fertilized eggs in stadium 8–16 cells (collected 2 h after spawning and washed with 0.1% methylene blue solution) were added within 22 ml medium (composition of the medium according to guideline, OECD, 2013a, 2013b) and 22 µl sample extract. Eggs were transferred to 24 well plates, one egg in 2 ml per well, including plate control. One positive control (PC; 3,4-dichloranilin, 4 mg/l), one negative control (NC; medium), and one solvent control (SC; DMSO) were included per replicate, with ten eggs per control. A total of three replicates were performed. The embryos were incubated at 26 °C in well plates with gas-permeable foil. They were evaluated every 24 h up to 120 hpf for lethal and sublethal effects. According to the OECD guideline, a test was classified as valid if at least 30% of the PC showed lethal effects and not more than 10% of the NC (OECD, 2013a, 2013b). All data presented the validity criteria. The effects were defined according to the study of von Hellfeld et al. (2020).

### Acute immobilization test with *Daphnia magna*

The present study performed the daphnia-immobilization test as a limit test according to the OECD guideline 202 (OECD, 2004). The *Daphnia magna Straus* were obtained from the Institute for Environmental Research culture at the RWTH Aachen University (the original daphnids are from stock cultures at the University of Sheffield; Agatz et al., 2012; Simon et al., 2015). At the start of the test, the neonates (younger than 24 h) were sieved and added directly to the test solution. The test medium was prepared according to the guideline (OECD, 2004). The experiments were performed in three replicates, with four glass tubes per sample. Five neonates were introduced per glass tube, with 10 µl sample extract in 10 ml medium. The NC was the respective medium, and the SC was DMSO. The tubes were stored at 20 ± 1 °C and a 16/8-h light/dark photoperiod. After 24 and 48 h, the immobility was examined. Immobilization was considered when individuals did not move after 15 s and gentle agitation. The validity criteria (pH value variation of < 1.5 units, dissolved oxygen > 3 mg/l, and < 10% of immobilized daphnia in the NC and SC) were fulfilled for all replicates and samples.

### Algae growth inhibition test

The test was performed according to the OECD guideline 201 (OECD, 2011). The green algae (Chlorophyta) *Raphidocelis subspicata* (formerly: *Pseudokirchneriella subcapitata*) was originally obtained from the culture collection of algae (SAG Göttingen, Germany). The short-term algae culture was diluted 1:10, and its density was measured (required cell density: 5000 cells/ml). The highest concentration was Ref 2 with a dilution series 1:6. As SC, 0.1 ml DMSO was prepared and as blank a sample without algae. The fluorescent density of the samples was measured with the multi-well plate reader (Tecan infinite M200; Tecan Group Ltd., Switzerland). All plates were closed with parafilm and incubated for 72 h. Every 24 h, the well plates were mixed for 1 min at 150 rpm before the fluorescent density was measured. The pH value was measured every 24 h and should be 8.1 ± 0.2. Further validity criteria regarding growth rate and mean coefficient of variation (OECD, 2011) were fulfilled for all experiments.

### Ames fluctuation assay

The test was performed according to the DIN EN ISO guideline 11,350:2012–05 (DIN EN ISO, 2012) and the study of Reifferscheid et al. (2011). The amino acid-dependent strains TA 98 and TA 100 of *Salmonella typhimurium* (Strains obtained from the Deutsche Forschungsgemeinschaft, DFG, Bonn, Germany) were tested with and without S9 (rat liver homogenate). The samples were tested in three independent replicates.

Both bacterial strains were grown with sterile growth medium (9.4 g nutrient broth, 0.6 g NaCl in 0.4 l H_2_O) and sterile ampicillin in an overnight culture (incubator at 12 °C until incubation period, 8.5 h, 37 °C, 150 rpm). With 500 µl total volume, sample extracts and DMSO were diluted 1:50 in the medium. First, the bacteria were diluted according to their measured FAU (Formazine Attenuation Unit; TA 98: FAU 170, TA 100: FAU 45). The plates with bacteria suspension, samples, S9-mix, PC, and NC were incubated for 100 min at 37° C while shaking. After incubation, 2.5 ml of reversion indicator medium was added. Then, transfer to the 384-well plates with 50 µl/well each and 48 wells per concentration and control.

The highest concentration was Ref 40, and the lowest was Ref 1.75. Several evaluation approaches were combined to achieve the best results (Levy et al., 2019). Revertant colonies were counted, and the induction factor (IF) was calculated by dividing the median result at each concentration by the median result with the corresponding negative control (Keiter et al., 2006; Kosmehl et al., 2004; Seitz et al., 2008). Additionally, the concentration-dependent induction factor (CDI) was calculated to rank the mutagenicity in environmental samples because it is independent of the tested concentrations (Shuliakevich et al., 2022a).

### Micro-EROD assay

The test was performed according to the developed protocol of Schiwy et al. (2015a). The cell suspension (prepared according to the protocol by Schiwy et al., 2015a) was diluted to a cell density of 200,000 cells/ml. Afterwards, 50 µl of the suspension was filled into 96-well plates and incubated for 2 h (37 °C, 5% CO_2_, 95% humidity). A total of 6 concentrations of the samples (1:2 series) were prepared, with Ref 20 as the highest concentration. TCDD (2,3,7,8-tetrachlorodibenzo-p-dioxin) was prepared in medium 1:100, and 50 µl each was added to the cells. The exposed samples were incubated for 70 h (37 °C, 5% CO_2_, 97% humidity). Subsequently, the medium was aspirated in a dark environment, 100 µl of the ETX stock solution (see protocol) was added, and the samples were incubated for 30 min (37 °C, 5% CO_2_, 95% humidity). A total of 75 µl methanol were added into each well and shaken for 20 min at room temperature to stop the reaction. The fluorescence of the samples (production of resorufin) was measured with the multi-well plate reader (Tecan infinite M200; Tecan Group Ltd., Switzerland). One protein standard (ETX solution with BSA standard; see Schiwy et al., 2015a) with 7 concentrations was prepared and transferred to the plate. After that, 100 µl of the bicinchoninic acid (BCA) reagent mix (Pierce BCA Protein Assay Kit; Thermo Scientific, Germany) was added to all cells. The plates were incubated for 40 min at room temperature. Afterwards, the absorption was measured at 550 nm. The validity criteria (Schiwy et al., 2015a) were achieved for all experiments.

### Yeast estrogen screen

The Yeast estrogen screen (YES) assay was performed according to DIN EN ISO 19040–1:2018 (DIN EN ISO, 2018) and the study of Routledge and Sumpter (1996). The yeasts (cryo culture; originally from Deutsche Forschungsgemeinschaft, DFG, Bonn, Germany) were prepared as an overnight culture (ONC) with 500 µl SD-medium and 500 µl DO-medium (22 h, 30 °C while shaking). The samples were diluted 1:100 in ultrapure water. In 96-well plates, a 1:2 serial dilution with Ref 20 as the highest concentration was performed. The final cell solution (ONC and DO-Medium) was prepared, and the FAU was measured (42.5 – 57.5 FAU). The exposure Medium (SD-Medium, DO-Medium, CuSO_4_, Ampicillin, streptomycin) and the cell solution were given in 96-well plates and shaken for 30 s at 450 rpm. The optical density (OD) was measured at 600 nm, and the plates were incubated for 18 h at 30 °C (covered with a gas-permeable film). After the incubation, the cell density was measured (600 nm). The lacZ reaction mixture was prepared and transferred to new 96-well plates with the samples. The OD was measured immediately at 580 nm. The plates were incubated for 1 h at 30 °C under agitation. The OD was measured at 580 nm and 540 nm. The validity criteria (DIN EN ISO, 2018) were fulfilled for all samples and all replicates.

### Data analysis

The data analysis was performed in an RStudio environment (Version: 2022–04-22 ucrt) by using the scripting language R (Version: 4.2.0) (R Core Team, 2019). Several packages in RStudio have been used: “compositions”, “psych”, “car”, “dplyr”, “ggplot2”, “pgirmess”, “survival”, and “PMCMRplus”. At first, the three river systems (perennial, ephemeral rivers, and the Iishana system) were tested for normal distribution with logarithm transformed data by applying the Shapiro–Wilk test, which is suitable and delivers good results for small sample sizes. Subsequently, a Levene test to check the differences of variances in case of a normal distribution. The not normally distributed data with unequal variances were tested with the Kruskal–Wallis test for significant differences between the three systems. The normally distributed data with unequal variances, i.e., the Ames assay, were also tested with the Kruskal–Wallis-Test. This test is suitable for testing non-normally distributed data and comparing the mean values of several groups without the requirement of a specific distribution form. The Friedman test was applied to gain knowledge of the differences between sampling sites. As a post hoc test, the Wilcoxon test was carried out. Differences between the Iishana and the perennial rivers were tested with the Mann–Whitney-U-test (95% confidence interval), because of unrelated and not normally distributed data and a small sample size. A two-way ANOVA and a post-hoc Tukey test were applied for the Ames assay to test for significant differences. With a* p*-value < 0.05, the results were defined as significant.

## Results

### Fish embryo toxicity test

**Fig. 2:**
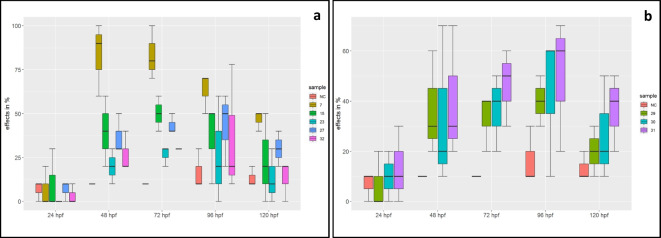
Observed effects of **a** the Iishana system and **b** the perennial rivers

Several sublethal effects, such as chorion deformations and edema, could be observed in the Iishana system, the Kunene River, and the Kavango River. The embryotoxic and teratogenic potential was similar in all systems, and the most observed effects were pericardium and yolk sack edema (see Fig. 2a, b, and Supplementary Data 2). After 48 h, all samples showed more than 10% effects (10 to 100% effects), except samples 15, 23, and 32. All samples showed a decrease in effects after 96 h. The striking effects were an affected, slightly decomposed chorion, pectoral fin underdevelopment, and blood congestion. In particular, at sites 7, 15, and 27 of the Iishana, these effects were noted, but at the Kunene and Kavango Rivers, these effects were detected less frequently (10% to 14% effects). Embryo malformation and development retardation were the most common effects at the perennial rivers. After 72 hpf 32% of the development retardations and failures were observed (Fig. 2a, b). However, there are no statistically significant differences between the Iishana system and the perennial rivers regarding the number of observed effects (*p* = 0.83).

No embryotoxic effects were detected in the ephemeral and perennial river systems, as the results were not significantly different from those of the negative control. The hatching success was equal in all three river systems compared with control embryos. Of the control group, 96% hatched until 120 hpf, and 99.6% of the exposed embryos.

### Daphnia acute immobilization test

**Fig. 3:**
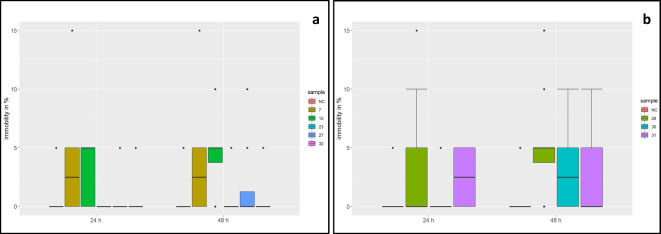
Immobilized *Daphnia magna* in **a** Iishana and **b** perennial rivers

The negative and solvent controls were identical, with 5% immobile daphnids in three replicates. An effect of acute immobility is defined as when > 10% of daphnids are immobilized. At the Iishana, sites 7, 15, and 27 and all sites at the perennial rivers show more than 10% effects. After 24 h, 6% of the daphnids exposed to Iishana water and 8% of the daphnids exposed to the water of the perennial rivers were immobilized. After 48 h, the immobilized daphnids increased to 9% at the Iishana and 13% at the perennial rivers (Fig. 3a, b). Samples from the Iishana and perennial rivers differ significantly in the number of immobilized daphnia (*p* = 0.049). The samples of the Iishana are not significantly different from the negative control, except for sites 7 (*p* = 0.0016) and 15 (*p* = 0.00004). However, the perennial waters significantly differ from the negative control (*p* < 0.05).

### Algae growth inhibition test

The growth inhibition study showed no effects on the algal species studied. The systems did not differ statistically from each other. Growth rates and inhibition rates can be found in Supplementary Data 3.

### Ames fluctuation assay

The investigation of the mutagenicity with the Ames fluctuation with *Salmonella typhimurium* strains TA98 and TA100 (± S9) showed effects for both river systems. All samples show an increasing number of wells of revertant growth over the concentration range compared to the NC (two-way ANOVA, *p* < 0.05). The visible reproducible increase in revertant counts compared to the negative control was significant for both strains for the sites 23, 27, and 29. At sites 15 and 32, the strain TA98 + and at sites 30 and 31, the strain TA98- were significant (see Supplementary Data 4). There are no statistically significant differences between the Iishana and the perennial rivers (*p* > 0.05).

**Fig. 4:**
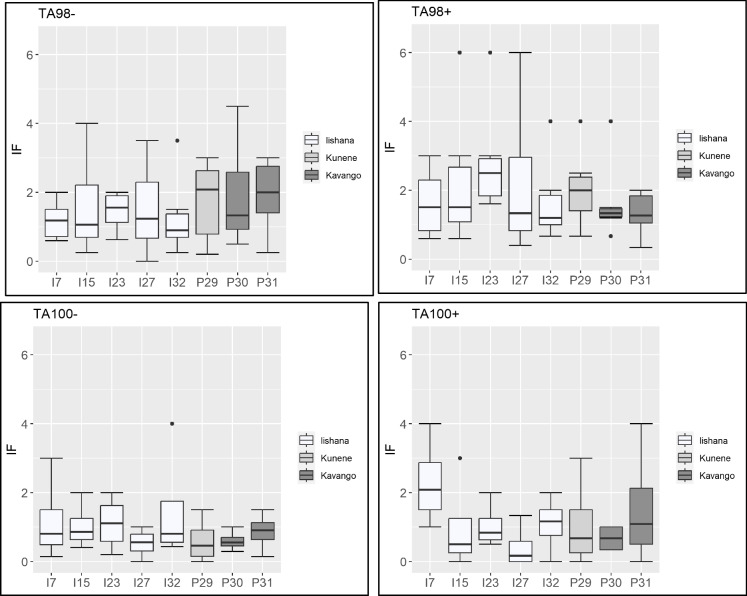
Distribution of induction factors (IF) evaluated by exposure of strain TA98 and TA100 ± S9 to the Iishana, and the Kunene and Kavango Rivers in six dilution steps and three replicates

Figure 4 shows the IF of both strains and all samples. A significant potential mutagenic activity is defined with an IF > 1.3 (Kosmehl et al., 2004). The strain TA98 (± S9) showed IF values > 1.3 in every sample. In contrast, investigation with the strain TA100 − showed at sites 27 and 30 IF values > 1.3 and with strain TA100 + at site 30. The IF of strain TA98 are higher than that of strain TA100 but not significantly different. TA98 + shows slightly higher IFs with a higher range (0.3 – 6) than TA98- (0 – 4.5). TA100 − and TA100 + have smaller ranges (both 0 – 4).

Seitz et al. (2008) introduced the CDI as an index value to compare environmental samples regarding their genotoxic potential. In Table 2, the concentrated-dependent induction factor (CDI) for the strains TA98 ± and TA100 ± for all samples are presented. The results show mutagenic potential in the strain TA98 (± S9). In contrast, no mutagenic potential was indicated for the strain TA100 (± S9).

**Table 2:** The average concentration-dependent induction factors (CDI) for the strains TA98 and TA100 ± S9 (*Salmonella typhimurium*) for the Iishana system and the Kunene and Kavango Rivers

	Iishana					Kunene	Kavango	
	7	15	23	27	32	29	30	31
TA98-	1.8	2.1	1.8	1.8	1.9	2.1	2.7	2.1
TA98 +	3.0	4.3	4.8	3.8	3.6	3.0	3.1	2.4
TA100 −	0.8	0.8	0.8	0.5	1.1	0.6	0.4	0.4
TA100 +	1.4	0.2	0.5	0.1	0.4	0.6	0.5	0.8

### Micro-EROD assay

Prior to the EROD assay, potential cytotoxicity was investigated. Subsequently, only the concentrations that did not show cytotoxicity (> 80% cell viability) were used for the EROD assay to exclude the possibility of masking the mechanism-specific effect. No cytotoxic effect could be detected for any samples so that a masking effect can be excluded. In addition, none of the samples tested showed dioxin-like potency in the EROD assay.

### Yeast estrogen screen

**Fig. 5:**
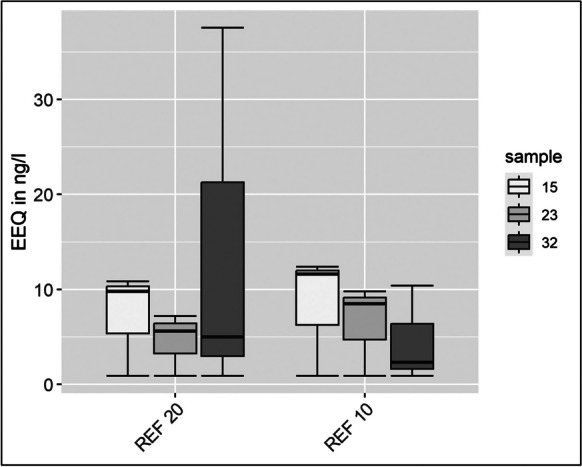
EEQ (17β-estradiol equivalents) in ng/l for the Iishana system

An endocrine-disrupting potential was found in samples 15, 23, and 32 of the Iishana system (Fig. 5). The concentrations of 10.89 ng/l ± 0.41 (sample 15), 5.62 ng/l ± 0.37 (sample 23), and 21.28 ng/l ± 1.19 (sample 32) were calculated for the two highest concentrations REF 20 and REF 10. The statistical analysis showed no significantly different results (p = 0.94) for samples 15 and 23. However, a different result could be observed for sample 32 with p-values of 0.007 and 0.015. In the lower concentrations, the measured values were below the limit of Quantification (LOQ). In all the other samples, the estrogenic activity was below the limit of detection (LOD) and the LOQ (for details see Supplementary Data 5).

## Discussion

All samples of the Iishana system and the perennial rivers showed > 10% sublethal and lethal effects per sample in the FET. The observed sublethal effects concerning the blood circulation system, such as edema and slow blood flow, can be caused by substances that bind to the Ah-receptor and act similarly to dioxin (Barron et al., 2004; Kais et al., 2017; Schiwy et al., 2015b). However, dioxin-like chemicals (DLCs) could not be detected in the micro-EROD assay, so they are probably excluded as possible causative agents. Further compounds, such as pesticides (Awoyemi et al., 2019), flame retardants (Parsons et al., 2019), nanomaterials (Shaw et al., 2016), and heavy metals (Taslima et al., 2022) can also cause edema. In the analyzed samples of the Iishana, concentrations of Cd (0.5 µg/l), Cr (3.94 µg/l), and Cu (37.33 µg/l) were detected in previous studies (Faulstich et al., 2023). Muzungaire et al. (2012) could detect 64.0 µg/l Fe and 9.0 µg/l Cu concentrations in the Kavango River. These metals could have caused pericardial edema and slow heart rate, even in small concentrations (Taslima et al., 2022). However, a comprehensive chemical analysis of the substances is to be performed, making it difficult to precisely identify the drivers of the toxic effects.

The pectoral fin underdevelopment could indicate that the fins lack blood supply. Von Hellfeld et al. (2020) observed fin underdevelopment caused by histone deacetylase (HDAC) inhibitors. Different HDACs were linked to a changed skeletal development in mammals, but the underlying functions of HDACs are comparable to the findings in fish embryos in the present study. In this study, the effect of an affected chorion appeared mostly after 48 hpf and 72 hpf at the Iishana. The metabolism of zebrafish embryos differs over time, and 72 hpf is the most suitable time for the use of the zebrafish, concerning the stability and viability of the embryos (Dhillon et al., 2019; Kais et al., 2017; Schiwy et al., 2015b). The embryos in affected chorions were mostly able to hatch, suggesting a substance that attacks the chorion but does not cause coagulation of the embryo. The affection and slight decomposition of the chorion can be caused by substances that cannot pass the chorion. During the hours after fertilization, the chorion changes its permeability (Pelka et al., 2017). If effects decrease over time, then the substance has either been used up, or biotransformation has taken place, and the hatched fish have degraded the substance.

Logan et al. (2023) identified several negative impacts of nanofibers, such as increasing apoptosis and the neutrophil response for the embryonic development of fish. Microplastic fragments and fibers have been detected in the Iishana system (Faulstich et al., 2023) and the blood stasis as a result of neutrophilic reactions could also be detected in the tested embryos. Therefore, it cannot be excluded that nanofibers may have negatively affected the embryonic development of *Danio rerio*.

In addition to the effects on fish embryos, acute effects on algae growth could also be observed. As mentioned before, heavy metals, such as Cd, Cr, and Cu, could be detected in the investigated samples (Faulstich et al., 2023). These analyzed heavy metals can also influence the observed toxicological effects on algae. For example, Ni and Cu affect the survival and the photosynthetic energy storage capacities of algae (Bossuyt & Janssen, 2004). In the Iishana system, 17.7 µg/l of Ni and 37.3 µg/l of Cu were found (Faulstich et al., 2023). In addition to its effects on fish embryos and algae, heavy metals negatively affect daphnia. Zn and Cu, for example, are likely more toxic for algae than daphnia (Ardestani et al., 2014). This study found stronger effects on daphnia than on algae, on the one hand, this could indicate that the metal concentrations are too low to cause effects in algae. On the other hand, interactions between metals and microorganisms could reduce the metal content in the water and cause less effects on algae (Priya et al., 2022).

The Iishana system has shown up to 30% effects on daphnia mobility. The immobility of daphnia could result from disseminated pollutants, for example microplastics (Samadi et al., 2022), heavy metals (Yuan et al., 2020), or antibiotics (Yisa et al., 2023). The perennial Rivers Kunene and Kavango showed no significant toxic effects on daphnia. These findings are in line with other publications. For example, investigations on the Cértima River in Portugal and the Sebou River in Morocco also showed no negative impact on *Daphnia magna* (Serpa et al., 2014; Koukal et al., 2004).

Several studies could prove a negative impact of wastewater treatment plants on the toxicity for the aquatic organisms (Ra et al., 2008; Shuliakevich et al. 2022c). The study area has a wastewater treatment plant near Outapi, a small town between site 23 and site 7 (Liehr et al., 2016). Brooks et al. (2006) describe the influence of effluent discharges on ephemeral systems. Flood events in the CEB can discharge wastewater and pollutants into the Iishana system. Major flood events occur regularly in the region, most recently in 2011 and 2013 (Arendt et al., 2021). In 2019, only low rainfall was recorded in the Iishana system, which could cause slow flooding. Besides wastewater, open landfill sites could be a source of toxic substances that cause effects in *Daphnia magna*. Wichmann et al. (2006) investigated a landfill site with open combustion and discovered effects < 20% in the test with *Daphnia magna*. Near Oshakati, between site 27 and site 32, is a landfill site with open combustion (Faulstich et al., 2023), whose effluents could be responsible for the effects found in this study, especially at site 32, which is close to the landfill site.

Besides the water bodies, suspended solids of the Iishana are also contaminated with metals: 4.9 µg/g Cd, 83.7 µg/g Cr, 166.9 µg/g Cu, 52.6 µg/g Ni, 82.7 µg/g Pb, 90.2 µg/g Sr, and 122.8 µg/g Zn (Faulstich et al., 2023). Contaminated particles can cause toxic effects on filter feeders such as Daphnids. On the one hand, xenobiotics are continuously dissolved from the particles; on the other hand, the particle-bound fraction can become available within the body of particle-feeding organisms. Consequently, this can lead to unexpectedly high tissue concentrations (Weltens et al., 2000). Therefore, further examining suspended solids for toxicological effects is reasonable.

Endocrine-disrupting compounds, such as 17α-ethinylestradiol (EE2) and 17β-estradiol (E2). are hormonally active, even at low concentrations, and are found in surface water and groundwater worldwide (Bistan et al., 2012; Klaic & Jirsa, 2022; Sumpter, 2005). The Iishana system and the perennial rivers Kunene and Kavango showed EEQ concentrations up to 37.55 ng/l. One reason for these high concentrations of EEQ could be the input of various wastewaters in case of floods and surface runoff from settlements, roads, and agriculture (Burkhardt-Holm, 2010). These high concentrations up to 40 ng/l, such as in the Iishana system, are commonly found in wastewater. Murk et al. (2002) detected with the YES assay up to 317 pmol EEQ/l (~ 141.1 ng/l) in untreated wastewater and 4 pmol EEQ/l in surface water (~ 1.8 ng/l). Kidd et al. (2007) demonstrated that 5–6 ng/l EEQ lead to a collapse of whole fish populations. Wolf et al. (2022) detected EEQ concentrations up to 2.7 ng/l in a small German river and detected that heavy rainfall events influence the input of endocrine compounds into the aquatic system. Kunz et al. (2015) reported concentrations of EEQ up to 9.4 ng/l in European surface waters. In South Africa, in Pretoria and Cape Town, they could not detect estrogenic potential in drinking water samples with the YES assay. Still, the T47D-KBluc bioassay delivered low estrogenic activities with EEQ values between 0.002 to 0.114 ng/l (van Zijl et al., 2017). These measurements from other studies and systems show that the measured concentrations of EEQ in surface waters in this study are unusually high. Aneck-Hahn et al. (2012) observed estrogenic activities in the South African Limpopo province. They discovered a connection between estrogenic activities and metal concentrations, as metals can bind the estrogen receptor alpha (ERα) and bold the binding of 17β-estradiol (Darbre, 2006). 17β-estradiol is released into the environment by agricultural runoff and animal excretions and was found in several studies (He et al., 2022; Liu et al., 2019; Perondi et al., 2020). Also, plastic compounds could be a source of estrogenic potential (Chen et al., 2019b). Some studies indicate an accumulation and adsorption of toxic contaminants, such as polychlorinated biphenyls (PCBs), bisphenol A (BPA), and heavy metals, on microplastics (Aragaw & Mekonnen, 2021; Vo & Pham, 2021; Wang et al., 2019). Microplastics were found in all three systems, and water and sediment samples in the Iishana system showed elevated metal concentrations (Faulstich et al., 2022 & 2023). It is unlikely that the present microplastics or the elevated metal concentrations caused the high EEQ concentrations up to 37.55 ng/l. Rather, there seems to be a source that releases these estrogenic substances. EDCs have already been found in surface waters in Namibian dams (Faul et al., 2014). Faul et al. show that EDCs were mainly found in water bodies close to urban activities and a high population density, such as Windhoek. Since similar conditions prevail in the Iishana system, it is possible that EDCs may also be found in surface waters there.

The Calux assay has often replaced the YES assay due to the partially missing transferability of yeasts to the human organism (Iuele et al., 2022; Nascimento et al., 2021). Nevertheless, the YES assay is still a good indicator for detecting endocrine potential in environmental samples and is used in aquatic systems in Southern Africa (Aneck-Hahn et al., 2006; Archer et al., 2020; Kasonga et al., 2021).

In addition to evaluating the estrogenic potential of the samples, potential mutagenicity was also investigated with the two strains of *Salmonella typhimurium* (TA98 and TA100). The results showed that the *Salmonella typhimurium* strain TA98 was more sensitive than TA100, resulting in a significant reproducible increase in revertant counts referred to the revertant number in the negative control in all samples of the Iishana. The strain TA100 only showed visible differences regarding the revertants. The number of revertants for the strain TA98 ± is slightly smaller than the spontaneous revertant control values (20–50 revertants) in the literature (Mortelmans & Zeiger, 2000; Tejs, 2008). For the strain TA100 ± , the control values (75–200 revertants) are significantly higher than those in this study. However, the measured revertant counts were significantly different from the NC. The strain TA98 is more sensitive and can detect frameshift mutations (Kosmehl et al., 2004). A low cell density of the strain TA100 and fewer targets for base substitution cause a lower sensitivity than the strain TA98 (Reifferscheid et al., 2011). Iji et al. (2021) analyzed the surface water of a stream in South Africa (Mpumalanga Province) with the Ames test to gain knowledge of the genotoxic potential. The IF of strain TA98 was > 1.5, and of strain TA100 > 1.7 (Iji et al., 2021). The metabolic activation by the rat liver S9-mix had a marginal effect on the mutagenic potential. All tested samples in this study have IF values > 1.3 for the strain TA98. Both strains show slightly higher IF values with the S9 mix and have a higher mutagenic potential. For a quantitative interpretation of genotoxicity and mutagenicity data, reference points are missing, like a benchmark dose. The dose–response relationship still needs to be evaluated (Menz et al., 2023). Therefore, it is difficult to quantitatively assess the genotoxicity and mutagenicity of the investigated river systems. Nevertheless, it is known that heavy metals can cause mutagenicity. In a case study in India, Rajput et al. (2020) described that samples with a higher concentration of heavy metals lead to a higher mutagenicity. As described before, heavy metals were found in the Iishana and could cause mutagenic effects.

## Conclusion and outlook

This study is the first study in Namibia that investigated surface waters regarding their ecotoxicological potential. It is an important step towards a complementary water quality assessment of these hardly-researched waters. The main objective of this proof of concept study was to identify the ecotoxicological potential of the Iishana system and the two neighboring systems, Kunene and Kavango. Several effects were detected in the investigated samples. Acute toxicity was detected in fish and daphnia, while freshwater algae showed few effects. The investigated river systems differ concerning the observed acute toxicity. The perennial rivers show fewer effects in the FET but more on the immobilization of daphnia than the Iishana. Common endpoints included a slightly decomposed chorion, pectoral fin underdevelopment, and blood congestion. Strong endocrine effects up to 40 ng EEQ/l were investigated and a significant mutagenic potential was identified for the strain TA98 ± .

The demonstrated ecotoxicological effects in the studied aquatic systems seriously affect water bodies and their ecosystems. Toxic substances, EDCs, and microplastics (Faulstich et al., 2022) threaten the ecosystem of the Iishana. Demonstrated acute toxicity to the daphnia and zebrafish may result in reproductive disruption, affecting the population size of primary and secondary consumers. There are several negative effects of estrogenic and estrogen-like compounds on endocrine systems, reproductive outcomes, and reproductive health of the population (Campbell et al., 2006; Hecker & Hollert, 2011; Woodruff, 2011). Estrogenicity may cause damage to the reproductive organs, thus also negatively affecting populations. When aquatic pollution affects keystone species, such as daphnia and algae, biodiversity is threatened. A loss of biodiversity in the Iishana, the Kunene and Kavango Rivers can reduce fish populations, which are important for the local food supply. The water of the Iishana is used as drinking water (Faulstich et al., 2023). This study showed that the Iishana water has toxic effects and it cannot be excluded that these will be transferred to humans, then this is a high risk for the local population and human health could be endangered by consuming Iishana water. Therefore, appropriate measures are needed to improve water quality, reduce toxicity, and ensure the use of the Iishana water as a water resource. Addressing acute effects, endocrine disruptions, and mutagenic effects in a river basin requires a comprehensive approach, considering various potential sources and factors. Applied measures could address five different aspects: (i) wastewater treatment plants (WWTP) improvement, (ii) landfill site management, (iii) green infrastructure, (iv) sustainable agriculture practices, and (v) remediation technologies.

The treatment technologies in wastewater treatment plants could be upgraded by advanced oxidation processes, membrane filtration, or activated carbon adsorption to enhance the removal of contaminants, including heavy metals and organic pollutants (Klaic & Jirsa, 2022). The installation of activated carbon filters at strategic points to adsorb and remove organic pollutants, mitigating acute toxicity and endocrine disruption.

Implementing monitoring systems that detect fluctuations in pollutant concentrations could enable rapid response to unexpected discharges and allow the optimization of treatment processes. Existing contaminated sites should be remediated to prevent the further release of pollutants (Koda et al., 2020). The adoption of sustainable waste management practices, such as recycling and waste-to-energy technologies, could minimize the generation of pollutants in the first place. Green infrastructure and natural filtration systems, such as riparian buffers, wetlands, and vegetated swales filter pollutants and improve water quality (Chen et al., 2019a; Saravanan et al., 2021). New management practices in agriculture minimize the runoff of agrochemicals and fertilizers into water bodies and reduce reliance on chemical pesticides, thereby minimizing the introduction of harmful substances into the environment. This includes contour plowing, cover cropping, and precision agriculture (Bai et al., 2019). The exploration of in situ remediation technologies, such as phytoremediation and biostimulation, can treat contaminated areas within the river basin without significant disturbance (Sharma et al., 2023).

Implementing these measures in a coordinated manner can contribute to the reduction of acute toxicity, endocrine disruptions, and mutagenic effects in a river basin. Sustainable management of the river basin, safeguarding both environmental and human health, is crucial. Therefore, regular reassessment and adaptation of strategies based on ongoing monitoring and research findings are necessary for long-term success.

Although the approach used in this study, a combination of several bioassays, has already been published in several studies, it delivers results for a region where these analyses have not been documented. To the authors’ knowledge, in Namibia, there are no studies concerning the ecotoxic potential of surface waters. Identifying toxic compounds is inevitable to estimate the state of the ecosystem. This study could prove the acute toxicity of the Iishana system, the Kavango, and the Kunene Rivers. Statements on chronic exposure cannot yet be made. Further investigations, for example testing the pre-filtered suspended solids, could help identify the relevant substances causing these effects.

Therefore, more effects may be detectable when using more sensitive test systems or other organisms likely more adapted to arid and semi-arid regions (Lahr, 1997). Increasing the exposure times for the endpoints mobility and growth reduction could cause the achievement of more effects. In the environment, organisms are exposed to potentially toxic substances for long durations. These long exposure times can be recreated in the laboratory. Harmful effects often occur after prolonged exposure to pollutants, since the environmental samples are also exposed to the potentially toxic substances for a longer period of time. Long-term toxicity tests, which simulate chronic exposure for up to several weeks or months based on the life cycle of the test organism, can be helpful in assessing long-term effects. For acute toxicity, reproduction can be examined as a “long-term” test for *Daphnia Magna* (OECD, 2012) and the early life stage test for *Danio rerio* (OECD, 2013b).

One decade ago, ecotoxicology was a relatively new science in South Africa (Wepener & Chapman, 2012), but it has developed rapidly, and nowadays, numerous studies can be found on ecotoxicological risk assessment of aquatic systems in the SADC region, with large differences between countries (Eijsackers et al., 2020; Selwe et al., 2022). Future investigations concerning freshwater quality should link chemical and bioanalytical information and quantify cause-effect relationships (Altenburger, 2019). Overall, managing and mitigating acute toxicity in surface waters is vital for maintaining the ecological integrity of aquatic ecosystems and ensuring the sustainable use of water resources for various human and environmental needs. For a holistic study of the Iishana ecosystem, a chemical analysis, and the analysis of sediments and suspended solids for toxic effects would complete the ecotoxicological assessment.

### Supplementary information

Below is the link to the electronic supplementary material.

Supplementary file1 (DOCX 28 KB)

## Data Availability

Data will be made available on request. Additional data are properly cited and referred to in the reference list.
