# Supplementary material for: Ecotoxicological evaluation of surface waters in Northern Namibia
Source: Environ Monit Assess. 2024 Apr 17;196(5):456. doi: 10.1007/s10661-024-12613-2 (PMC11024038; doi:10.1007/s10661-024-12613-2)
Supplement: Supplementary file 1 — Supplementary file1 (DOCX 28 KB) [file 10661_2024_12613_MOESM1_ESM.docx]

**Ecotoxicological evaluation of surface waters in Northern Namibia**

*Environmental Monitoring and Assessment*

Faulstich, L.; Wollenweber, S.; Reinhardt-Imjela, Ch.; Arendt, R.; Schulte, A.; Hollert, H.; Schiwy, S.

Freie Universität Berlin, leona.faulstich@fu-berlin.de

**Supplementary data 1**

General information of the samples: table differentiated by sampling site

| **name** | **system** | **water depth [m]** | | **depth of view [m]** | **color** | **temperature [°C]** |
| --- | --- | --- | --- | --- | --- | --- |
| **site 7** | Iishana | 0.2 | 0.07 | | dark grey | 22.44 |
| **site 15** | Iishana | > 0.8 | 0.40 | | grey | 24.52 |
| **site 23** | Iishana | 0.5 | 0.07 | | green, yellow | 32.11 |
| **site 27** | Iishana | > 0.7 | 0.08 | | grey | 28.37 |
| **site 32** | Iishana | > 0.9 | 0.09 | | green | 25.98 |
| **site 29** | Kunene | > 0.7 | 0.02 | | grey | 32.15 |
| **site 30** | Kavango | > 0.7 | > 0.70 | | transparent | 31.27 |
| **site 31** | Kavango | > 0.7 | > 0.70 | | transparent | 31.14 |

**Ecotoxicological evaluation of surface waters in Northern Namibia**

*Environmental Monitoring and Assessment*

Faulstich, L.; Wollenweber, S.; Reinhardt-Imjela, Ch.; Arendt, R.; Schulte, A.; Hollert, H.; Schiwy, S.

Freie Universität Berlin, leona.faulstich@fu-berlin.de

**Supplementary data 2**

Observed sublethal and lethal effects of zebrafish embryos. Data represent mean and standard deviation of % effects observed during acute fish embryo toxicity test.

|  | **hours post fertilisation (hpf)** | | | | | | | | |
| --- | --- | --- | --- | --- | --- | --- | --- | --- | --- |
|  | **24 hpf** | | | **48 hpf** | | | **72 hpf** | | |
|  | **Iishana** | **Kunene** | **Kavango** | **Iishana** | **Kunene** | **Kavango** | **Iishana** | **Kunene** | **Kavango** |
| **development retardations & failures** |  |  |  |  |  |  |  |  |  |
| lack of somite formation | -- | -- | 1.7 ± 3.7 | -- | -- | -- | -- | -- | -- |
| lack of tail detachment | 0.7 ± 2.5 | -- | 1.7 ± 3.7 | -- | -- | -- | -- | -- | -- |
| missing spontaneous movements | 2 ± 5.4 | -- | -- | 6 ± 9.5 | 10 ± 14.1 | 15 ± 21.4 | -- | -- | -- |
| embryo malformation | -- | -- | 1.7 ± 3.7 | -- | -- | 1.7 ± 3.7 | -- | -- | 3.3 ± 4.7 |
| impaired/missing heart-beat | -- | -- | -- | -- | -- | 1.7 ± 3.7 | -- | -- | 1.7 ± 3.7 |
| impaired/missing blood flow | -- | -- | -- | -- | -- | 3.3 ± 4.7 | -- | -- | 3.3 ± 4.7 |
| blood congestion | -- | -- | -- | -- | -- | -- | 0.7 ± 2.5 | 10 ± 8.2 |  |
| pericardium edema | -- | -- | -- | 14.7 ± 12.6 | 10 ± 8.2 | 10 ± 5.8 | 24 ± 11.4 | 23.3 ± 4.7 | 23.3 ± 12.5 |
| yolk sack edema | -- | -- | -- | 5.3 ± 7.2 | 10 ± 8.2 | 3.3 ± 4.7 | 9.3 ± 10.6 | -- | 8.3 ± 6.9 |
| spine deformation | -- | -- | -- | 2 ± 5.4 | -- | -- | -- | -- | -- |
| impaired pectoral fin development | -- | -- | -- | -- | -- | -- | 1.3 ± 3.4 | -- | -- |
| fin deformation (no fin mobility) | -- | -- | -- | -- | -- | -- | -- | -- | -- |
| damaged chorion | -- | -- | -- | 0.7 ± 2.5 | -- | -- | -- | -- | -- |
| chorion deformation | 0.7 ± 2.5 | -- | -- | 14.7 ± 30.3 | -- | -- | 14 ± 29.6 | -- | -- |
| unnormal swimming behavior | -- | -- | -- | -- | -- | -- | -- | -- | -- |
|  |  |  |  |  |  |  |  |  |  |
| **hatching** |  |  |  |  |  |  |  |  |  |
| hatched | -- | -- | -- | -- | -- | -- | 2 ± 4.0 | -- | -- |
| not hatched | -- | -- | -- | -- | -- | -- | 94 ± 6.1 | 90 ± 0 | 90 ± 0 |
|  |  |  |  |  |  |  |  |  |  |
| **lethality** |  |  |  |  |  |  |  |  |  |
| coagulation | 3.3 ± 4.7 | 10 ± 8.2 | 10 ± 11.5 | 4 ± 6.1 | 10 ± 8.2 | 10 ± 11.5 | 4 ± 6.1 | 10 ± 8.2 | 10 ± 11.5 |

Table continued

|  | **96 hpf** | | | **120 hpf** | | |
| --- | --- | --- | --- | --- | --- | --- |
|  | **Iishana** | **Kunene** | **Kavango** | **Iishana** | **Kunene** | **Kavango** |
| **development retardations & failures** |  |  |  |  |  |  |
| lack of somite formation | -- | -- | -- | -- | -- | -- |
| lack of tail detachment | -- | -- | -- | -- | -- | -- |
| missing spontaneous movements | -- | -- | -- | -- | -- | -- |
| embryo malformation | -- | -- | 3.3 ± 4.7 | -- | -- | 3.3 ± 4.7 |
| impaired/missing heart-beat | -- | -- | 3.3 ± 4.7 | -- | -- | 3.3 ± 4.7 |
| impaired/missing blood flow | -- | -- | 3.3 ± 4.7 | -- | -- | 3.3 ± 4.7 |
| blood congestion | 0.7 ± 2.5 | 6.7 ± 9.4 |  | 1.3 ± 5.0 | -- | -- |
| pericardium edema | 20.7 ± 17.3 | 16.7 ± 17.0 | 11.7 ± 10.7 | 10.7 ± 13.9 | 6.7 ± 4.7 | 11.7 ± 10.7 |
| yolk sack edema | 1.3 ± 3.4 |  | 3.3 ± 4.7 | 2.7 ± 5.7 | -- | 3.3 ± 4.7 |
| spine deformation | 1.3 ± 3.4 | 3.3 ± 4.7 | 5 ± 7.6 | 0.7 ± 2.5 | -- | 1.7 ± 3.7 |
| impaired pectoral fin development | 4 ± 10.0 |  | 3.3 ± 7.5 | 0.7 ± 2.5 | -- | 1.7 ± 3.7 |
| fin deformation (no fin mobility) | 6.7 ± 10.1 | 3.3 ± 4.7 | 5 ± 7.6 | 6.7 ± 10.1 | 3.3 ± 4.7 | 5 ± 7.6 |
| damaged chorion | -- | -- | -- | -- | -- | -- |
| chorion deformation | 2.7 ± 10.0 | -- | -- | -- | -- | -- |
| unnormal swimming behavior | -- | -- | -- | 2 ± 5.4 | -- | -- |
|  |  |  |  |  |  |  |
| **hatching** |  |  |  |  |  |  |
| hatched | 83.3 ± 13.0 | 80 ± 16.3 | 70 ± 19.1 | 95.3 ± 24.5 | 90 ± 8.2 | 88.3 ± 10.7 |
| not hatched | 12.7 ± 12.4 | 10 ± 8.2 | 20 ± 17.3 | 0.7 ± 2.5 | -- | 1.7 ± 3.7 |
|  |  |  |  |  |  |  |
| **lethality** |  |  |  |  |  |  |
| coagulation | 4 ± 6.1 | 10 ± 8.2 | 10 ± 11.5 | 4 ± 6.1 | 10 ± 8.2 | 10 ± 11.5 |

**Ecotoxicological evaluation of surface waters in Northern Namibia**

*Environmental Monitoring and Assessment*

Faulstich, L.; Wollenweber, S.; Reinhardt-Imjela, Ch.; Arendt, R.; Schulte, A.; Hollert, H.; Schiwy, S.

Freie Universität Berlin, leona.faulstich@fu-berlin.de

**Supplementary data 3**

Specific growth inhibition in % (mean ± standard deviation) of the Iishana system, the Kunene, and the Kavango Rivers.

| **Iishana** | | | | | | | | | | | | | |
| --- | --- | --- | --- | --- | --- | --- | --- | --- | --- | --- | --- | --- | --- |
| **Conc mg/l** | **NC** | **PC** | | **3.2** | | **1.6** | | **0.8** | | **0.4** | | **0.2** | |
| **7** | | | | | | | | | | | | | |
| **growth rate**  **(mean ± sd) [%]** | 1.8 ± 0.1 | 1.6 ± 0.6 | | 1.9 ± 0.1 | | 1.8 ± 0.1 | | 1.8 ± 0.1 | | 1.9 ± 0.1 | | 1.9 ± 0.1 | |
| **inhibition [%]** | 0.0 | 9.9 | | -4.9 | | -2.4 | | -3.7 | | -5.3 | | -4.8 | |
| **normalized inhibition [%]** | 0.0 | 9.9 | | 0.0 | | 0.0 | | 0.0 | | 0.0 | | 0.0 | |
| **15** | | | | | | | | | | | | | |
| **growth rate**  **(mean ± sd) [%]** | 1.7 ± 0.1 | 1.6 ± 0.8 | | 1.9 ± 0.3 | | 2.0 ± 0.3 | | 2.0 ± 0.3 | | 2.0 ± 0.4 | | 2.0 ± 0.4 | |
| **inhibition [%]** | 0.0 | 5.9 | | -12.5 | | -16.4 | | -14.7 | | -17.1 | | -14.8 | |
| **normalized inhibition [%]** | 0.0 | 5.9 | | 0.0 | | 0.0 | | 0.0 | | 0.0 | | 0.0 | |
| **23** | | | | | | | | | | | | | |
| **growth rate**  **(mean ± sd) [%]** | 1.8 ± 0.1 | 1.7 ± 0.8 | | 2.0 ± 0.2 | | 2.0 ± 0.2 | | 2.0 ± 0.2 | | 2.0 ± 0.3 | | 1.9 ± 0.1 | |
| **inhibition [%]** | 0.0 | 5.8 | | -9.7 | | -10.5 | | -11.0 | | -12.4 | | -6.4 | |
| **normalized inhibition [%]** | 0.0 | 5.8 | | 0.0 | | 0.0 | | 0.0 | | 0.0 | | 0.0 | |
| **27** | | | | | | | | | | | | | |
| **growth rate**  **(mean ± sd) [%]** | 1.9 ± 0.1 | 1.8 ± 0.7 | | 2.0 ± 0.2 | | 2.0 ± 0.2 | | 2.0 ± 0.3 | | 2.0 ± 0.3 | | 1.8 ± 0.2 | |
| **inhibition [%]** | 0.0 | 7.5 | | -5.4 | | -4.9 | | -5.8 | | -2.2 | | 3.9 | |
| **normalized inhibition [%]** | 0.0 | 7.5 | | 0.0 | | 0.0 | | 0.0 | | 0.0 | | 3.9 | |
| **32** | | | | | | | | | | | | | |
| **growth rate**  **(mean ± sd) [%]** | 1.8 ± 0.1 | 1.7 ± 0.6 | | 2.0 ± 0.3 | | 2.0 ± 0.3 | | 2.0 ± 0.4 | | 2.0 ± 0.4 | | 2.0 ± 0.4 | |
| **inhibition [%]** | 0.0 | 4.8 | | -8.1 | | -9.1 | | -11.5 | | -9.9 | | -9.2 | |
| **normalized inhibition [%]** | 0.0 | 4.8 | | 0.0 | | 0.0 | | 0.0 | | 0.0 | | 0.0 | |
|  |  |  | |  | |  | |  | |  | |  | |
| **Perennial rivers** | | | | | | | | | | | | | |
| **Conc mg/l** | **NC** | | **PC** | | **3.2** | | **1.6** | | **0.8** | | **0.4** | | **0.2** |
| **29** | | | | | | | | | | | | | |
| **growth rate**  **(mean ± sd) [%]** | 1.9 ± 0.1 | | 1.6 ± 0.6 | | 1.9 ± 0.2 | | 1.9 ± 0.3 | | 1.9 ± 0.2 | | 1.9 ± 0.3 | | 1.9 ± 0.3 |
| **inhibition [%]** | 0.0 | | 16.9 | | -0.3 | | -0.9 | | 2.2 | | 0.1 | | 0.0 |
| **normalized inhibition [%]** | 0.0 | | 16.9 | | 0.0 | | 0.0 | | 2.2 | | 0.1 | | 0.0 |
| **30** |  | |  | |  | |  | |  | |  | |  |
| **growth rate**  **(mean ± sd) [%]** | 1.9 ± 0.1 | | 1.7 ± 0.5 | | 1.9 ± 0.2 | | 1.9 ± 0.2 | | 2.0 ± 0.3 | | 1.9 ± 0.3 | | 2.0 ± 0.3 |
| **inhibition [%]** | 0.0 | | 10.3 | | -3.0 | | -3.0 | | -5.7 | | -3.3 | | -5.0 |
| **normalized inhibition [%]** | 0.0 | | 10.3 | | 0.0 | | 0.0 | | 0.0 | | 0.0 | | 0.0 |
| **31** | | | | | | | | | | | | | |
| **growth rate**  **(mean ± sd) [%]** | 1.9 ± 0.1 | | 1.5 ± 0.7 | | 1.9 ± 0.3 | | 1.9 ± 0.3 | | 1.9 ± 0.3 | | 1.8 ± 0.3 | | 1.8 ± 0.3 |
| **inhibition [%]** | 0.0 | | 17.4 | | -2.7 | | -2.2 | | -1.7 | | 1.3 | | 1.2 |
| **normalized inhibition [%]** | 0.0 | | 17.4 | | 0.0 | | 0.0 | | 0.0 | | 1.3 | | 1.2 |

**Ecotoxicological evaluation of surface waters in Northern Namibia**

*Environmental Monitoring and Assessment*

Faulstich, L.; Wollenweber, S.; Reinhardt-Imjela, Ch.; Arendt, R.; Schulte, A.; Hollert, H.; Schiwy, S.

Freie Universität Berlin, leona.faulstich@fu-berlin.de

**Supplementary data 4**

Mutagenic potential of the samples (Iishana and perennial rivers) based on a visible reproducible increase in revertant counts at single dilution steps referred to the revertant number in the negative control. Significant differences (p-values) are presented in grey and visible differences in white. All samples were tested in six dilution steps and three independent replicates.

|  | **Iishana** | | | | | **Kunene** | **Kavango** | |
| --- | --- | --- | --- | --- | --- | --- | --- | --- |
|  | **7** | **15** | **23** | **27** | **32** | **29** | **30** | **31** |
| **TA98-** | 0.18 | 0.08 | 0.003 | 0.02 | 0.14 | 0.02 | 0.008 | 0.002 |
| **TA98+** | 0.4 | 0.02 | 0.0002 | 0.03 | 0.008 | 0.05 | 0.06 | 0.08 |
| **TA100-** | 0.18 | 0.08 | 0.29 | 0.08 | 0.17 | 0.08 | 0.08 | 0.08 |
| **TA10+** | 0.18 | 0.18 | 0.18 | 0.57 | 0.58 | 0.57 | 0.18 | 0.18 |

**Ecotoxicological evaluation of surface waters in Northern Namibia**

*Environmental Monitoring and Assessment*

Faulstich, L.; Wollenweber, S.; Reinhardt-Imjela, Ch.; Arendt, R.; Schulte, A.; Hollert, H.; Schiwy, S.

Freie Universität Berlin, leona.faulstich@fu-berlin.de

**Supplementary data 5**

Limit of Detection (LOD) and Limit of Quantification (LOQ) of the YES assay for all samples.

|  | **Replicate 1** | | **Replicate 2** | | **Replicate 3** | |
| --- | --- | --- | --- | --- | --- | --- |
|  | **LOD [ng/l]** | **LOQ [ng/l]** | **LOD [ng/l]** | **LOQ [ng/l]** | **LOD [ng/l]** | **LOQ [ng/l]** |
| **site 7** | 3.4 | 4.7 | 34.6 | 54.7 | 4.6 | 10.2 |
| **site 15** | 0.9 | 2.8 | 19.2 | 31.3 | 1.6 | 4.1 |
| **site 23** | 0.9 | 2.8 | 19.2 | 31.3 | 1.6 | 4.1 |
| **site 27** | 6.1 | 8.5 | 40.9 | 54.4 | 2.8 | 5.7 |
| **site 32** | 27.2 | 33.3 | 23.7 | 40.4 | 2.5 | 4.0 |
| **site 29** | 6.1 | 8.5 | 40.9 | 54.4 | 2.8 | 5.7 |
| **site 30** | 31.5 | 42.2 | 23.1 | 38.1 | 4.3 | 10.6 |
| **site 31** | 31.5 | 42.2 | 23.1 | 38.1 | 4.3 | 10.6 |
